# Assessing the Functional and Structural Properties of Peanut Meals Modified by Transglutaminase-Coupled Glycation

**DOI:** 10.3390/foods14111999

**Published:** 2025-06-05

**Authors:** Yan Liu, Tingwei Zhu, Fusheng Chen, Xingfeng Guo, Chenxian Yang, Yu Chen, Lifen Zhang

**Affiliations:** College of Food Science and Engineering, Henan University of Technology, Zhengzhou 450001, China; liuyan3345746684@outlook.com (Y.L.); guoxingfeng@haut.edu.cn (X.G.); xcyang@haut.edu.cn (C.Y.); loyyer@163.com (Y.C.); zhanglifen2013@hotmail.com (L.Z.)

**Keywords:** peanut meal, transglutaminase, glycation, functional properties, structure

## Abstract

To increase the added value of peanut meal (PM, protein content of 46.17%) and expand its application in food processing, cold-pressed PM was modified via transglutaminase (TGase)-coupled glycation to enhance its functional properties. The effects of the modification conditions (i.e., PM concentration, PM/glucose mass ratio, temperature, and time) on the functional properties of PM were investigated, and its structural properties were evaluated using water contact angle measurements, fluorescence spectroscopy, and Fourier-transform infrared spectroscopy. It was found that TGase-coupled glycation modification altered the secondary structure of PM and increased both the water contact angle and the surface hydrophobicity, thereby significantly affecting its functional properties. Additionally, superior emulsification, foaming, and oil-absorbing properties were achieved for the modified PM, which were named EPM, FPM, and OPM, respectively (specimens under different modification conditions). Notably, the emulsification activity of the EPM sample was enhanced by 69.8% (i.e., from 18.48 to 31.38 m^2^/g); the foaming capacity of the FPM specimen was increased by 84.00% (i.e., from 21.00 to 46.00%); and the oil-absorbing capacity of the OPM sample was enhanced by 359.57% (i.e., from 1.41 to 6.48 g/g protein).

## 1. Introduction

Peanut meal (PM) is a by-product of peanut oil extraction and can be classified as either hot-pressed or cold-pressed PM, depending on the processing method employed for oil extraction. As previously reported, cold-pressed PM demonstrates a low degree of protein denaturation, a high nutritional value, and a good application value [1]. The protein content of PM is ~50%, and its component proteins contain a large number of essential amino acids. The digestibility of peanut protein can reach up to 90%, and its nutritional value is similar to that of animal proteins, thereby indicating its potential as an animal protein substitute [2,3]. Currently, the majority of PM is employed to extract the peanut proteins for modification or direct usage through its addition to food products [4,5]. However, the protein content of PM is lower than that of peanut protein isolate, thereby resulting in a large gap between the functional properties of these two products and limiting the application of PM in food systems. It is therefore desirable to explore green, simple, and effective modification methods for PM to enhance its functional properties and promote the development of the peanut protein industry.

Current methods for protein modification mainly include physical, chemical, and biological enzymatic modification [6], wherein complex modifications have been demonstrated to produce proteins with improved functional properties [7,8]. For example, the glycation reaction between proteins and reducing sugars represents a promising approach for enhancing the functional properties of proteins due to its mild nature and the absence of additional chemicals [9,10]. Enzymatic modification is also widely used in the food industry owing to its strong specificity and high safety profile. In this context, transglutaminase (TGase) has attracted a great deal of attention owing to its safe and abundant nature [11]. Additionally, the emulsifying properties of modified soybean isolates obtained via a combination of glycation and TGase modification were found to be higher than those of the modified soybean isolates obtained using the two methods individually [12]. Furthermore, complex modifications lead to changes in the protein secondary structure and surface hydrophobicity, ultimately affecting their functional properties [13]. It is therefore important to study the structure–function relationships of modified proteins to broaden their applications in food processing.

During protein modification using the TGase and glycation approach, the reaction conditions, including the type of sugar donor, the reaction temperature, and the reaction time, are known to significantly affect the protein function and structure [14,15]. As a widely distributed monosaccharide in nature, glucose (Glu) exhibits good hydrophilicity and reducibility and contains several hydroxyl and aldehyde groups; therefore, it is often used as a glycosyl donor for protein modification [16,17]. The modification of PM via TGase-coupled glycation with a Glu glycosyl donor would be expected to enhance the functional properties of PM and increase the added value of peanut meal. However, the effects of the modification conditions on the functional properties and structural characteristics of PM during this complex modification process have yet to be investigated.

Thus, in this study, Glu and TGase are used to modify cold-pressed PM via the TGase-coupled glycation protocol. The effects of the modification conditions (i.e., Glu concentration, reaction time, temperature, and PM concentration) on the solubility, emulsification, foaming, water-holding, and oil-absorbing characteristics of PM are investigated. Then, the structural properties of the modified PM (i.e., contact angle, fluorescence spectroscopy, Fourier-transform infrared (FTIR) spectroscopy, and secondary structure) were also analyzed to interpret the differences in functional properties of modified PM. This study aims to enhance the functional properties of PM through TGase-coupled glycation to expand its application in the food industry.

## 2. Materials and Methods

### 2.1. Materials

The PM (protein content 46.17%, N × 6.25) employed herein was purchased from Qingdao Long Unk Foods Co., Ltd. (Qingdao, China). Glu was obtained from Shengda Food Additives Co., Ltd. (Qingdao, China), while TGase (activity unit, 120 U/g) was purchased from Dongsheng Biotechnology Co., Ltd. (Taixing, China). The soybean oil was purchased from a local supermarket (Zhengzhou, China).

### 2.2. Preparation of the Modified PM (MPM)

PM and Glu were initially mixed in the desired mass ratio (1:1, 3:2, 2:1, 3:1, 4:1, 5:1, or 6:1, *w*/*w*) and then dissolved in a 0.1 mol/L phosphate-buffered saline (PBS) solution (pH 7.8) to adjust the PM concentration to 5, 10, 15, or 20%. Subsequently, TGase (0.5 g/100 g protein) was added, and the mixed solution was stirred at room temperature for 1 h. The obtained mixtures were then heated at different temperatures (35, 40, 45, 50, and 55 °C) for the desired time (3.0, 3.5, 4.0, 4.5, or 5.0 h). Finally, the resulting mixtures were freeze-dried for 72 h and stored at 4 °C until required for evaluation.

### 2.3. Determination of the MPM Solubility

According to the method described by Liu et al. [18], a standard curve was constructed using albumin as the standard protein. More specifically, each sample (0.12 g, without Glu) was accurately weighed, water (10 mL) was added, and the resulting mixture was stirred at room temperature for 1 h prior to subsequent centrifugation at 10,000 rpm for 15 min. The protein concentration in the supernatant was determined using the biuret method, while the solubility of the MPM was calculated using Equation (1).

(1) $$\mathrm{Solubility}\;(\%)=\frac{\mathrm{Protein}\;\mathrm{content}\;\mathrm{in}\;\mathrm{supernatant}}{\mathrm{Total}\;\mathrm{protein}\;\mathrm{content}\;\mathrm{in}\;\mathrm{sample}}\times 100$$

### 2.4. Evaluation of the Emulsifying Activity Index (EAI) and the Emulsion Stability Index (ESI)

The emulsifying activity index (EAI) and the emulsion stability index (ESI) were measured using the method described by Naik et al. [19] with some modifications. More specifically, soybean oil (1.0 mL) was mixed with the sample solution (9.0 mL, 2.5% m/v) and sheared at 15,500 rpm for 1 min (FM200, FLUKO, Berlin, Germany). Aliquots (50 μL) of the sample were extracted from the bottom of the solution at times of 0 and 10 min, then added to a 0.1% solution of sodium dodecylsulphate (SDS, 5 mL). The absorbance was then measured at 500 nm, using the 0.1% SDS solution as a blank. The EAI and ESI were calculated using Equations (2) and (3), respectively.

(2) $${\mathrm{EAI}\;(m}^{2}/g)=\frac{2\times 2.303\times A_{0}\times n}{c\times \varphi \times L\times 10,000}$$

(3) $$\mathrm{ESI}\;(\min)=\frac{A_{0}}{A_{0}-A_{10}}\times t$$

where 2.303 is the light extinction coefficient, *c* is the weight of protein per volume (g/mL); *n* is the dilution factor (dimensionless); *φ* is the oil volume fraction of the emulsion (dimensionless); *L* is the thickness of the cuvette (*L* = 1 cm); *t* is a time interval (*t* = 10 min); and *A*_0_ and *A*_10_ are the absorbance readings at 0 and 10 min, respectively.

### 2.5. Evaluation of the Foaming Capacity (FC) and Foaming Stability (FS)

Referring to the method of Zhang et al. [20], the sample (0.400 g, without Glu) was dissolved in water (10 mL) and sheared at 12,500 rpm for 2 min (FM200, FLUKO, Germany). The foam volume was then recorded as *V*_1_, and after a further 30 min, the foam volume was recorded once again as *V*_2_. Subsequently, the foaming capacity (FC) and foaming stability (FS) were calculated using Equations (4) and (5), respectively.

(4) $$\mathrm{FC}\;(\%)=\frac{V_{1}}{10}\times 100$$

(5) $$\mathrm{FS}\;(\%)=\frac{V_{1}}{V_{2}}\times 100$$

### 2.6. Determination of the Water-Holding Capacity (WHC) and the Oil-Absorbing Capacity (OAC)

Referring to the method of Shen et al. [21], the sample (0.12 g, without Glu, *W*_1_/*O*_1_) was weighed into centrifuge tubes of known masses (*W*_2_/*O*_2_), and either water or soybean oil (5 mL) was added to provide the corresponding dispersions or solutions. The obtained samples were then allowed to stand at room temperature for 30 min. After subsequent centrifugation at 5000 rpm for 10 min, the supernatants were discarded, and the tubes were weighed (*W*_3_/*O*_3_). The water-holding capacity (WHC) and oil-absorbing capacity (OAC) were calculated for each sample according to Equations (6) and (7), respectively.

(6) $$\mathrm{WHC}=\frac{W_{3}-W_{2}-W_{1}}{W_{1}}$$

(7) $$\mathrm{OAC}=\frac{O_{3}-O_{2}-O_{1}}{O_{1}}$$

### 2.7. Determination of the Particle Size

According to the method of Gul et al. [22] with some modification, each sample (0.400 g) was dissolved in distilled water (10 mL) and stirred at room temperature for 1 h prior to centrifugation. The particle size distribution within the supernatant was determined using a particle size analyzer (Nano-ZS90, Malvern PANalytical, Malvern, UK), with an equilibration time of 30 s, a scattering angle of 173°, and a refractive index of 1.33. Each sample was measured in triplicate.

### 2.8. Determination of the Surface Hydrophobicity (H_0_)

According to the method of Ghorbani et al. [23] with some modification, the surface hydrophobicities (H_0_) of the samples were determined using the 8-aniline-1-naphthalene sulfonic acid (ANS) fluorescent dye. Initially, the protein dispersions were diluted using distilled water to give concentrations of 0.05–0.40 mg/mL (Glu mass excluded). Subsequently, an 8 mM ANS solution (20 μL) was added to an aliquot (4 mL) of each diluted sample. Measurements were conducted using fluorescence spectrophotometry (Cary Eclipse, Agilent, USA) with an excitation wavelength of 390 nm, an emission wavelength of 470 nm, and a slit size of 5 nm. The initial slope of the fluorescence intensity versus the protein concentration curve was calculated using linear regression analysis to give the value of H_0_.

### 2.9. Determination of the Water Contact Angle

Referring to the method of Chen et al. [24] with some modification, each sample (200 mg) was pressed using a tablet press for 1 min to obtain a thin tablet. Subsequently, a drop of pure water was added to the surface, and the droplet shape was photographed and analyzed using a contact angle meter (DSA25, KRÜSS, Hamburg, Germany).

### 2.10. FTIR Spectroscopy

Referring to the method of Wang et al. [25] with some modification, the sample for analysis was mixed with anhydrous KBr in a precise mass ratio of 1:100, and the FTIR absorption spectra were measured in the range of 4000–400 cm^−1^ (Tensor II, Bruker, Karlsruhe, Germany).

### 2.11. Statistical Analysis

All tests were conducted in triplicate from the same PM batch, and the results are presented as the mean ± the standard deviation (SD). Analysis of variance (ANOVA) and Duncan’s test were used to identify significant differences (*p* < 0.05), using SPSS Statistics 20 software (SPSS Inc., Chicago, IL, USA).

## 3. Results and Discussion

### 3.1. Evaluation of the MPM Solubility

**Figure 1:**
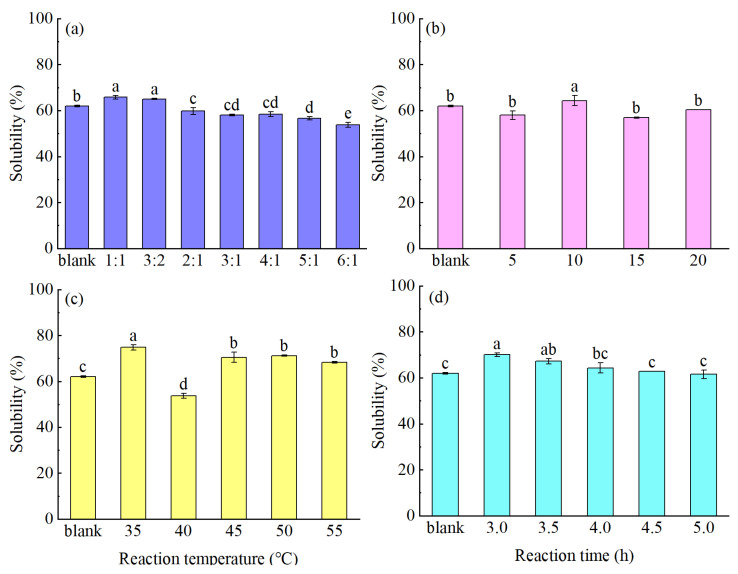
Effects of different reaction conditions (**a**) PM/Gluratio; (**b**) PM concentration; (**c**) temperature; (**d**) reaction time on the solubility of the PM (different letters indicate significant differences (*p* < 0.05) between the samples).

**Figure 2:**
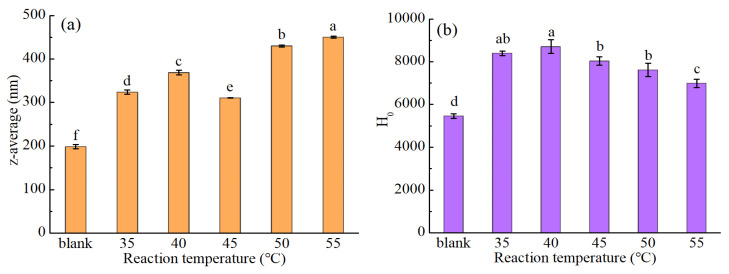
Effects of the reaction temperature on (**a**) the particle size (z-average) and (**b**) the surface hydrophobicity (H_0_) of the MPM (PM/Glu = 3:2, 10% PM, 4 h heating). (The different lowercase letters indicate significant difference (*p* < 0.05) between each sample).

Protein solubility plays a crucial role in defining the other functional properties of proteins and is of particular importance in the context of food processing [26]. As shown in Figure 1a–d, suitable modification conditions were defined for enhancing the solubility of the MPM protein. More specifically, using a PM/Glu mass ratio of 3:2, a PM concentration of 10%, a reaction temperature of 35 °C, and a reaction time of 3.0 h, the MPM solubility reached 70.04%, representing a 13.00% increase compared with that of the original PM. Since the addition of TGase alone tends to reduce the protein solubility, PM was modified using a TGase-coupled glycosylation protocol in the current study. During the modification process, the introduction of Glu increased the solubility of MPM under certain conditions. For example, it can be seen from Figure 1c that the MPM solubility tended to decrease with an increasing temperature, although the lowest value was observed at 40 °C, rather than at higher temperatures. It is well known that the solubility of a protein is related to its particle size and H_0_. In general, a larger particle size leads to a reduced solubility, while a high H_0_ disrupts the hydrophilic/lipophilic balance and leads to a reduced solubility [27]. To account for the significantly reduced solubility observed at 40 °C, the effects of temperature on the protein particle size and H_0_ value were further evaluated. As shown in Figure 2, the particle size of the MPM obtained at 40 °C was larger than those at 35 and 45 °C, and the MPM prepared at 40 °C also exhibited the maximum H_0_ value. This indicates that TGase was most active at 40 °C, thereby promoting protein cross-linking to produce macromolecules. Upon further increasing the reaction temperature, the glycosylation reaction was favored, which gradually introduced additional hydrophilic groups (-OH) onto the protein surface, ultimately leading to a decrease in H_0_ and an increased solubility.

### 3.2. Emulsifying Properties of the MPM

**Figure 3:**
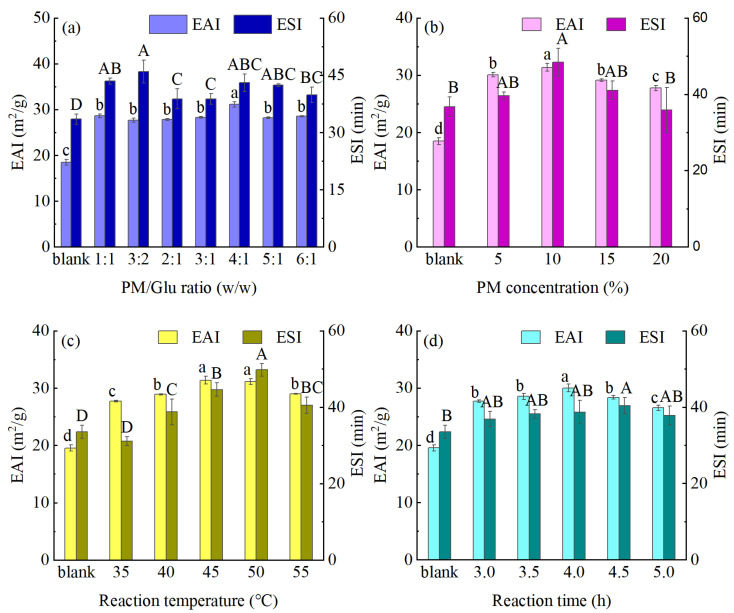
Effects of different reaction conditions (**a**) PM/Gluratio; (**b**) PM concentration; (**c**) temperature; (**d**) reaction time on the emulsifying properties of the PM (different capital and lowercase letters indicate significant differences (*p* < 0.05) in each indicator between the samples).

The emulsifying properties of proteins can be evaluated using their EAI and ESI values. More specifically, the EAI represents the ability of a protein to disperse the oil phase in the aqueous phase, while the ESI dictates the stability of the emulsion with regard to resisting creaming, flocculation, and coalescence [28]. The observed variations in the emulsifying properties of the MPM prepared via TGase-coupled glycosylation are shown in Figure 3. As indicated, the EAI values of the MPM specimens were significantly higher than that of the blank PM. Using a PM/Glu mass ratio of 4:1, a PM concentration of 10%, a temperature of 50 °C, and a modification time of 4 h, the EAI of the resulting MPM reached 31.38 m^2^/g, which is 69.80% higher than that of the blank group (18.48 m^2^/g). Previously, Ding et al. [12] investigated the effect of the Maillard reaction and TGase cross-linking on the functional properties of isolated soybean proteins and found that the introduction of sugar led to an increase in the number of protein hydrophilic groups, along with an increase in the EAI. However, Li et al. [29] found that emulsion stability was related to the length of the sugar chain, and glycosylation of Glu enhanced the EAI of soy peptides but had no effect on the ESI, whereas dextran-modified soy peptides exhibited higher ESI. As shown in Figure 3a, varying the PM/Glu ratio did not have a significant effect on the emulsion stability, perhaps due to the type of sugar. More specifically, Glu has a short sugar chain and provides a weak force, which is insufficient to improve the stability of the emulsion system. In contrast, in this study, TGase modification led to unfolding and polymerization of the protein molecules, which could subsequently encapsulate oil droplets more effectively and increase both the EAI and ESI values of the protein. In the current system, it was found that both the EAI and ESI values tended to increase and then later decrease with an increasing PM concentration, reaction temperature, and reaction time (Figure 3b–d), which may be attributed to the increased particle size of the protein molecules over a longer reaction time. As previously reported, a larger particle size slows the protein diffusion rate, thereby reducing the emulsification characteristics [30]. Overall, the MPM exhibiting the most pronounced emulsification characteristics (denoted EPM) was obtained under a PM/Glu mass ratio of 4:1, a PM concentration of 10%, a reaction temperature of 50 °C, and a reaction time of 4.0 h.

### 3.3. Foaming Characteristics of the MPM Samples

**Figure 4:**
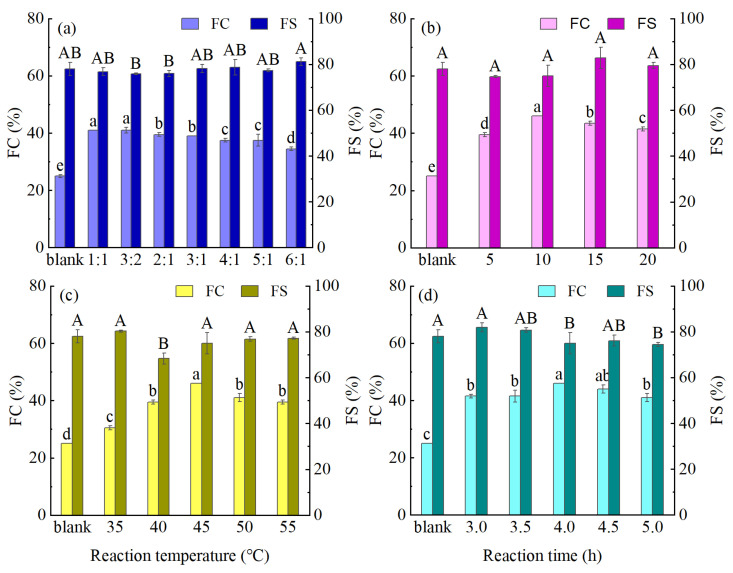
Effects of different reaction conditions (**a**) PM/Gluratio; (**b**) PM concentration; (**c**) temperature; (**d**) reaction time on the foaming properties of the PM (different capital and lowercase letters indicate significant differences (*p* < 0.05) in each indicator between the samples).

Foaming characteristics play a crucial role in defining the sensorial attributes of foods; therefore, the effects of the modification conditions on the FC and FS characteristics of the prepared MPM specimens were investigated. As indicated in Figure 4, following the TGase-coupled glycosylation protocol, the FC of the MPM was significantly higher than that of the blank group, although the FS did not change significantly. In general, the FC depends on the ability of a protein to be quickly adsorbed at the gas–liquid interface during churning to reduce the interfacial tension, whereas the FS is determined by the strength and viscoelasticity of the interfacial membrane formed between the interfacial proteins [31]. It was therefore considered that TGase-coupled glycosylation modification may unfold the PM structure, thereby exposing the hydrophobic groups and leading to an increase in the protein H_0_ (Figure 2b). Consequently, the protein can rapidly adsorb at the interface, which in turn enhances the FC [32]. As can be seen from Figure 4a, the FC gradually decreased with a decreasing sugar content. This was due to the introduced sugar chains increasing the protein hydrophilicity and solubility and the enhanced adsorption of the glycosylated proteins at the gas–liquid interface, which ultimately led to an increased FC [33]. Additionally, as shown in Figure 4b–d, upon increasing the PM concentration, reaction temperature, and reaction time, the FC showed a tendency to increase and then decrease. TGase can cause the protein to unfold and the hydrophobic groups to be exposed, resulting in more hydrophobic group binding sites on the protein surface [34]. At the initial stage of the reaction, the hydrophobic and hydrophilic groups on the surface of the protein increased under the joint action of TGase and glycosylation, and the protein can be quickly adsorbed to the gas–liquid interface to enhance the FC. With the further deepening of the reaction, the polymer of larger molecular weight was formed under the action of TGase. The resulting MPM was not easily adsorbed at the gas–liquid interface, leading to a reduction in the FC. However, a good FC does not necessarily indicate a good FS. More specifically, as illustrated in Figure 4, after protein modification, the FC significantly improved, whereas the FS exhibited the opposite trend. Dong et al. [35] observed similar results in their study on the effects of modifications on the FC and FS characteristics of proteins. In this case, the MPM exhibiting the optimal foaming properties (denoted FPM) was produced using a PM/Glu mass ratio of 3:2, a PM concentration of 10%, a reaction temperature of 45 °C, and a reaction time of 4.0 h with an FC of 84.00%.

### 3.4. WHC and OAC Evaluations for the Prepared MPM Specimens

**Figure 5:**
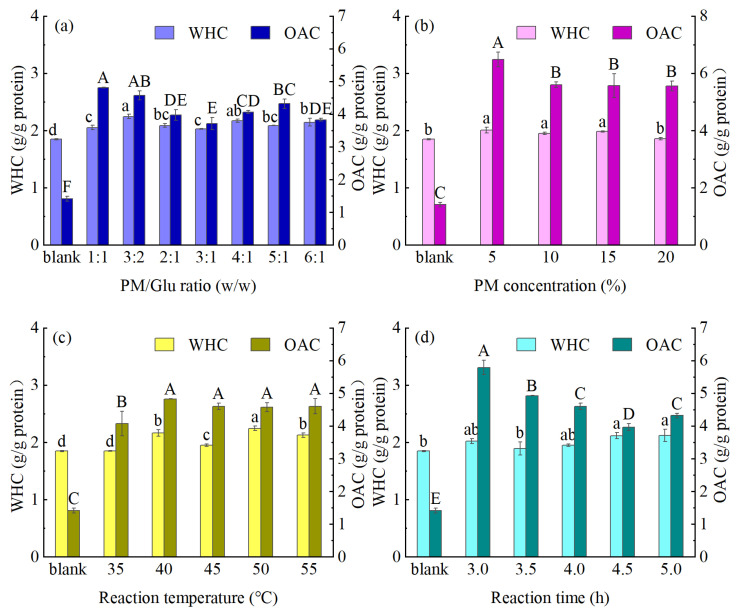
Effects of different reaction conditions (**a**) PM/Gluratio; (**b**) PM concentration; (**c**) temperature; (**d**) reaction time on the WHC and OAC properties of the PM (different capital and lowercase letters indicate significant differences (*p* < 0.05) in each indicator between the samples).

The WHC and the OAC determine the strength of the protein–water/oil interactions and affect the texture and quality characteristics of food products [36]. Figure 5 illustrates the effects of different modification conditions on the WHCs and OACs of the prepared MPM specimens. Generally, it was found that TGase-coupled glycosylation had a positive effect on both the WHC and the OAC of PM. Zhang et al. [37] found that peanut proteins treated with TGase alone had higher OAC but no significant effect on WHC. In contrast, in the present study, more hydrophilic groups were introduced by the glycosylation reaction, which led to an improvement in the WHC of MPM as well. However, the overall improvement in OAC was the most significant. Using a PM/Glu ratio of 3:2, a PM concentration of 5%, a reaction temperature of 50 °C, and a reaction time of 3.0 h, the MPM exhibited a good OAC of 6.48 g/g protein, which represents an increase of 359.57% compared to the PM (1.41 g/g protein). This can be accounted for by considering that the OAC is related to the number of hydrophobic amino acid residues on the protein surface, in addition to its hydrophobic amino acid content [21]. TGase-coupled glycosylation modification likely exposed additional PM hydrophobic groups, leading to an enhanced protein–oil binding, thereby increasing the OAC. Therefore, the PM exhibiting the highest OAC (denoted OPM) was obtained using the optimized reaction condition mentioned above.

### 3.5. Structural Characteristics of the MPM Specimens

**Figure 6:**
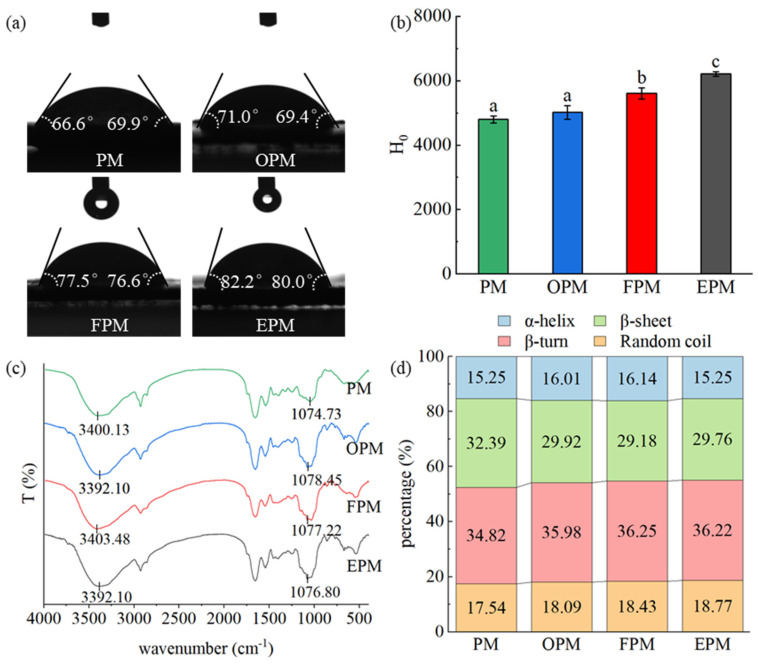
Properties and structural characteristics of the PM, OPM, FPM, and EPM species. (**a**) Water contact angle measurements, (**b**) hydrophobicity evaluations (Different lowercase letters indicate significant difference (*p* < 0.05) between each sample), (**c**) FTIR spectra, and (**d**) secondary structure contents.

The functional properties of proteins are related to their structural properties. Thus, to evaluate this in the current study, the water contact angle, H_0_, and secondary structure were examined for the three optimized MPM specimens (i.e., OPM, FPM, and EPM), using the unmodified PM as a control. As shown in Figure 6a,b, the EPM exhibited a larger contact angle and a higher H_0_ than the PM, OPM, and FPM samples. As mentioned previously, the H_0_ of a protein represents the extent to which its hydrophobic groups are exposed on the surface, while the water contact angle indicates the surface wettability of the protein. Thus, increases in both the H_0_ and the water contact angle indicate an increase in the exposed hydrophobic groups from the protein chain [38,39]. Previously, it has been shown that although unfolding of the peanut protein structure following TGase treatment exposes additional hydrophobic groups, glycosylation introduces hydrophilic groups (-OH), thereby leading to a reduction in the water contact angle and the H_0_ value [40,41]. In the case of the OPM specimen, neither the water contact angle nor the H_0_ changed significantly compared to those of the PM, probably because the hydrophilic groups introduced on the surface of the OPM balanced the exposed hydrophobic groups. In contrast, the increased H_0_ values observed for the FPM and EPM samples were likely due to the exposure of more abundant hydrophobic groups compared to the number of introduced hydrophilic groups, thereby leading to higher H_0_ values for the EPM and FPM species compared to that of the unmodified PM.

Further analysis of the protein structure by FTIR spectroscopy revealed that the IR spectra of the OPM, FPM, and EPM samples exhibited enhanced peak intensities at 1075 cm^−1^ compared with that observed for the PM specimen. In addition, the characteristic bands observed at 3700–3200 and 1100–1000 cm^−1^ corresponded to the stretching vibrations of the -OH and C–O groups [42]. The enhanced peak intensity at 1075 cm^−1^ may therefore arise due to changes in the C–O stretching vibration upon the introduction of -OH groups. Additionally, the amide I band was analyzed to study changes in the secondary structure of the PM protein [43]. As shown in Figure 6c,d, the main components of the PM were β-sheets and β-turns, with contents of 32.39 and 34.82%, respectively. Following TGase-coupled glycosylation, the β-sheet contents decreased while the β-turn contents increased in the OPM, FPM, and EPM samples. Additionally, the irregular curl content of the original PM was 17.54%, while the corresponding contents for the OPM, FPM, and EPM samples were 18.09, 18.43, and 18.77%, respectively. The shift from β-sheets to β-turns and irregular curl structures indicates that the protein molecular chain is moderately unfolded, which is conducive to the exposure of additional hydrophobic groups [44]. The EPM specimen exhibited the largest irregular curl content, which was attributed to a greater number of exposed hydrophobic groups, as supported by the higher water contact angle and H0 value for this protein.

## 4. Conclusions

In the current study, it was demonstrated that glycosylation coupled with transglutaminase (TGase) modification enhanced the functional properties of peanut meal (PM). In particular, the reaction temperature, PM concentration, and PM/sugar ratio showed varying degrees of influence on the solubility, emulsification, foaming, and water/oil holding capacity characteristics of the modified PM samples (MPM). Using a reaction temperature of 50 °C, a PM concentration of 10%, and a PM/Glu mass ratio of 4:1, the PM specimen exhibiting the greatest emulsifying properties (denoted EPM) was obtained. Meanwhile, the EPM specimen exhibited a high water contact angle (82.2°), an increased β-turn content (36.22%), and a reduced β-sheet content (29.76%). Furthermore, the PM specimen exhibiting the greatest foaming capacity was produced at a reaction temperature of 45 °C, a PM concentration of 10%, a reaction time of 4.0 h, and a PM/Glu mass ratio of 3:2 (denoted FPM). Moreover, using a reaction temperature of 50 °C, a PM concentration of 5%, a reaction time of 3.0 h, and a PM/Glu ratio of 3:2, the PM specimen exhibiting the highest oil-absorbing capacity (denoted OPM) was obtained. TGase-coupled glycation modification alters the secondary structure of PM and exposes more hydrophobic groups along with the introduction of sugar chains, which further influences its functional properties. In this study, the TGase-coupled glycosylation modification can increase both hydrophilic and hydrophobic groups on the surface of the protein to make up for the deficiency of single modification. However, it is undeniable that peanut allergen data were missing in this study, and further studies on the effect of TGase-coupled glycosylation modification on peanut allergy are needed in the future. Meanwhile, in the next study, the modified PM was applied to HIPEs to further validate the possibility of its application in food products such as salad dressings. Overall, this complex modification protocol can effectively improve and tune the functional properties of PM, with the overall aim of expanding its application in the food industry.

## Data Availability

The original contributions presented in this study are included in the article. Further inquiries can be directed to the corresponding authors.
